# Discovery of a Small-Molecule Inhibitor Targeting the Biofilm Regulator BrpT in *Vibrio vulnificus*

**DOI:** 10.4014/jmb.2406.06052

**Published:** 2024-09-20

**Authors:** Wonwoo Choi, Hojun Lee, Qiyao Wang, Ye-Ji Bang, Sang Ho Choi

**Affiliations:** 1 National Research Laboratory of Molecular Microbiology and Toxicology, Department of Agricultural Biotechnology, Seoul National University, Seoul 08826, Republic of Korea; 2 Center for Food and Bioconvergence, Seoul National University, Seoul 08826, Republic of Korea; 3 State Key Laboratory of Bioreactor Engineering, Shanghai Frontiers Science Center of Optogenetic Techniques for Cell Metabolism, East China University of Science and Technology, Shanghai 200237, P.R. China; 4 Shanghai Engineering Research Center of Maricultured Animal Vaccines, Shanghai 200237, P.R, China; 5 Department of Biomedical Sciences, Seoul National University College of Medicine, Seoul 03080, Republic of Korea; 6 Department of Microbiology and Immunology, Seoul National University College of Medicine, Seoul 03080, Republic of Korea; 7 Institute of Endemic Diseases, Seoul National University Medical Research Center, Seoul 03080, Republic of Korea; 8 Research Institute of Agriculture and Life Science, Seoul National University, Seoul 08826, Republic of Korea

**Keywords:** *Vibrio vulnificus*, biofilm, small-molecule inhibitor, high-throughput screening, BrpT, transcription regulation

## Abstract

*Vibrio vulnificus*, an opportunistic human pathogen, employs biofilm formation as a key survival and virulence mechanism. BrpT, a transcriptional regulator, is essential for *V. vulnificus* biofilm development by regulating the expression of biofilm-related genes. In this study, we aimed to identify a small molecule inhibitor of BrpT to combat *V. vulnificus* biofilm formation. High-throughput screening of 7,251 compounds using an *Escherichia coli* reporter strain carrying the arabinose-inducible *brpT* gene and a BrpT-activated promoter fused to the *luxCDABE* operon identified a hit compound, BTI (BrpT Inhibitor). BTI potently inhibited BrpT activity in *V. vulnificus* (EC_50_ of 6.48 μM) without affecting bacterial growth or host cell viability. Treatment with BTI significantly reduced the expression of the BrpT regulon and impaired biofilm formation and colony rugosity in *V. vulnificus*, thus increasing its susceptibility to antibiotics. In vitro biochemical analyses revealed that BTI directly binds to BrpT and inhibits its transcriptional regulatory activity. The identification of BTI as a specific inhibitor of BrpT that effectively diminishes *V. vulnificus* biofilm formation provides a promising foundation for the development of novel anti-biofilm strategies, with the potential to address the growing challenge of antibiotic resistance and improve the treatment of biofilm-associated infections.

## Introduction

*Vibrio vulnificus* is a gram-negative, opportunistic human pathogen that inhabits coastal waters and brackish environments worldwide [1]. It can cause severe wound infections, gastroenteritis, and life-threatening septicemia, with mortality rates exceeding 50% in susceptible individuals [2-4]. The ability of *V. vulnificus* to form biofilms on various surfaces, including seafood, is a critical factor contributing to its environmental persistence, antibiotic resistance, and pathogenesis [5, 6]. Biofilms are multicellular communities of bacteria encased in a self-produced extracellular matrix that provides protection against environmental stressors, host immune defenses, and antimicrobial agents [7-9]. The formation of biofilms by *V. vulnificus* enhances its survival and transmission, posing significant challenges for the prevention and treatment of infections [5, 10]. Therefore, the development of innovative approaches to combat *V. vulnificus* biofilms, such as small molecule inhibitors of biofilm regulators, is crucial for mitigating the public health risks associated with this pathogen.

The regulation of biofilm formation in *V. vulnificus* is a complex process involving multiple signaling pathways and transcriptional regulators [11]. One of the key regulators is BrpT, a transcription factor that directly activates the expression of numerous genes essential for biofilm development [12]. The BrpT regulon includes those within the *brp* locus (*brpABCDFHIJK*) responsible for exopolysaccharide (EPS) synthesis and secretion, *brpN* encoding an acetyltransferase involved in EPS biosynthesis, the *cabABC* operon encoding a matrix protein (CabA) and its secretion system (CabBC), and *cabH* encoding a surface attachment protein CabH [12-16]. Deletion of *brpT* significantly impairs *V. vulnificus* biofilm formation and rugose colony morphology, highlighting its pivotal role in governing the biofilm program [17]. Furthermore, BrpT has been shown to autoregulate its own expression, creating a positive feedback loop that amplifies its regulatory effects [11]. Given the central role of BrpT in controlling the expression of multiple biofilm-related genes, targeting BrpT activity represents an attractive strategy to inhibit *V. vulnificus* biofilm formation and mitigate its associated threats to human health.

Small molecules that disrupt biofilm formation by interfering with key regulatory pathways have emerged as promising alternatives to conventional antibiotics [18]. Such anti-biofilm agents offer several advantages, including reduced selective pressure for resistance and enhanced antibiotic susceptibility of biofilm-dwelling bacteria [6]. While several small-molecule inhibitors targeting biofilm formation in various bacterial pathogens have been identified [19], specific inhibitors of *V. vulnificus* biofilm regulators have not been reported to date.

In this study, we aimed to identify and characterize small-molecule inhibitors of BrpT to develop a novel strategy for combating *V. vulnificus* biofilm formation. We conducted a high-throughput screening of 7,251 compounds and identified a small molecule, BTI (BrpT Inhibitor), that significantly inhibited the BrpT activity in *V. vulnificus*. Without affecting bacterial growth or exhibiting host cell cytotoxicity, BTI effectively reduced the expression of the BrpT regulon, and thus impaired biofilm formation in *V. vulnificus*. Biochemical analyses demonstrated that BTI directly binds to BrpT and diminishes its transcriptional regulatory activity. Our findings establish BTI as a promising anti-biofilm agent and provide new insights into the regulation of biofilm formation in *V. vulnificus*.

## Materials and Methods

### Bacterial Strains, Plasmids, and Culture Conditions

The strains and plasmids used in this study are listed in Table 1. *Escherichia coli* strains were grown in Luria-Bertani (LB) medium at 37°C, while *V. vulnificus* strains were grown in LB medium supplemented with 2.0% (*w/v*) NaCl (LBS) at 30°C. The *Vibrio fischeri* minimal medium containing glycerol (VFMG) [50 mM Tris-HCl pH 7.2, 50 mM MgSO_4_, 300 mM NaCl, 10 mM KCl, 0.33 mM K_2_HPO_4_, 18.5 mM NH_4_Cl, 10 mM CaCl_2_, and 32.6 mM glycerol] was used for biofilm formation [20]. Antibiotics were added to the media at the following concentrations when required: kanamycin (100 μg/ml) and chloramphenicol (3 μg/ml). *V. vulnificus* JN111 [14], carrying *dcpA* encoding a diguanylate cyclase under the control of an arabinose-inducible promoter (P_BAD_) on the chromosome [21, 22], was used to manipulate intracellular c-di-GMP levels. Bacterial growth was monitored spectrophotometrically at absorbance 600 nm (*A*_600_). HeLa cells originated from the American Type Culture Collection [23] were maintained at 37°C with 5% CO_2_ in Dulbecco’s Modified Eagle’s Medium (DMEM) containing 10% fetal bovine serum (FBS).

### High-Throughput Screening and Hit Validation

A small-molecule library containing 7,251 compounds was generously provided by the Korea Chemical Bank (http://www.chembank.org). The compounds were dissolved in dimethyl sulfoxide (DMSO) and screened at a final concentration of 20 μM. The *E. coli* DH5α reporter strain, co-transformed with pJN1602 (expressing *brpT* under the control of P_BAD_) [17] and pWW2202 (carrying the promoterless *luxCDABE* operon (*lux* operon hereafter)) [24] fused to the BrpT-activated promoter P_*cabH*_ [15]), was used for the high-throughput screening. The reporter strain was cultured in LB medium containing 0.0002% (*w/v*) L-arabinose to an *A*_600_ of 0.5, and then 100 μl of the culture was transferred to each well of a 96-well microtiter plate (BD Falcon, USA) containing the compounds or 2% DMSO (negative control). After 4 h of incubation at 37°C with shaking, luminescence and bacterial growth (*A*_600_) were measured using a microplate reader (Tecan, Switzerland). Relative luminescence units (RLU) were calculated by dividing the luminescence by the *A*_600_ value. Hit compounds showing a statistically significant RLU reduction (greater than 49%) were further validated using *V. vulnificus* reporter strains: JN111 (parental wild-type) and JN111 *ΔbrpT* (*brpT* deletion mutant), both carrying pWW2201 (containing the promoterless *lux* operon fused to the BrpT-regulated promoter P_*cabA*_). The *V. vulnificus* reporter strains were cultured in LBS medium containing 0.02% (*w/v*) L-arabinose to an *A*_600_ of 0.5, and then 100 μl of the culture was transferred to each well of a 96-well microtiter plate (BD Falcon) containing the compounds or 2% DMSO (negative control). After 4 h of incubation at 30°C with shaking, luminescence and bacterial growth (*A*_600_) were measured using a microplate reader (Tecan).

### Determination of the EC_50_ of BTI

To determine EC_50_ (the concentration of BTI inhibiting cellular BrpT activity by 50%), the *V. vulnificus* reporter strain, JN111 containing pWW2201, was exposed to various concentrations of BTI. Luminescence and *A*_600_ of the reporter strain were measured after 4 h incubation at 30°C using a microplate reader, and RLUs were calculated. The cellular activity of BrpT was expressed using the RLU observed in the absence of BTI as 100% and RLU observed in *ΔbrpT* as 0%. The EC_50_ was calculated by plotting the relative cellular activity of BrpT versus the BTI concentrations using GraphPad Prism 9.0 (GraphPad Software, USA).

### Lactate Dehydrogenase (LDH) Assay

To examine the cytotoxicity of BTI, monolayers of HeLa cells grown in a 96-well tissue culture plate (Nunc, Denmark) were treated with various concentrations of BTI, 1% DMSO (control), or wild-type *V. vulnificus* at a multiplicity of infection (MOI) of 10. After 3 h incubation at 37°C, LDH activities in the supernatant were measured using a cytotoxicity detection kit (Roche, Germany) as described previously [25].

### Quantitative Reverse Transcription-PCR (qRT-PCR)

*V. vulnificus* strains grown to an *A*_600_ of 0.5 were treated with 100 μM BTI or 1% DMSO (control) and further incubated to the log phase (*A*_600_ of 2.0). Total RNA was then isolated using an RNeasy Mini Kit (Qiagen, USA). For qRT-PCR, the concentrations of the total RNAs were measured with a NanoDrop One spectrophotometer (Thermo Fisher Scientific, USA). cDNA was synthesized using an iScript cDNA synthesis kit (Bio-Rad, USA). Real-time PCR amplification of the cDNA was performed with the CFX97 real-time PCR detection system (Bio-Rad) with pairs of specific primers (Table S1). Relative levels of the transcripts were calculated by using the *rrsH* expression level as the internal reference for normalization [26].

### Biofilm Formation Assay

Biofilm formation was quantified using the crystal violet staining method as described previously [27]. Briefly, *V. vulnificus* cultures grown to an *A*_600_ of 0.8 were diluted to an *A*_600_ of 0.05 in VFMG supplemented with 0.01%arabinose and various concentrations of BTI or 1% DMSO (control). The diluted cultures (200 μl) were transferred to each well of a 96-well polystyrene microtiter plate (Nunc) and incubated statically at 30°C for 24 h. The biofilms were stained with 1% (*w/v*) crystal violet solution for 15 min, washed with phosphate-buffered saline (PBS), and the absorbed dye was eluted with ethanol. The absorbance of the eluted dye was measured at 570 nm (*A*_570_) using a microplate reader.

### Colony Morphology Analysis

For colony morphology analysis, 2 μl of *V. vulnificus* cultures grown to an *A*_600_ of 0.8 were spotted onto VFMG agar plates containing 0.02% arabinose and either 100 μM BTI or 0.1% DMSO (control). The plates were incubated at 30°C for 24 h, and the colony morphology was observed using a Stemi 305 stereomicroscope (Zeiss, Germany) equipped with an Axiocam 105 color camera (Zeiss).

### Protein Purification and Microscale Thermophoresis (MST) Analysis

The 6xHis-tagged BrpT protein was overexpressed in *E. coli* BL21 (DE3) using pSH1819 (carrying the *brpT* gene on pET-28a(+) (Novagen, USA) [17]) and purified by affinity chromatography using Ni-NTA agarose (Qiagen) as previously described [28]. The purified BrpT protein was labeled with the His-Tag Labeling Kit RED-tris-NTA 2nd Generation (NanoTemper Technologies) according to the manufacturer's protocol. The labeled BrpT (at a final concentration of 10 nM) was mixed with a serial dilution of BTI (500 μM (2^-1^ mM) to 15.2 nM (2^-16^ mM)) in BTI binding buffer (50 mM Tris-HCl pH 7.5, 150 mM NaCl, 10 mM MgCl_2_, 0.05% Tween-20, 5%Glycerol). After a 10min incubation at room temperature, the samples were loaded into Premium Coated Capillaries (NanoTemper Technologies) and analyzed using a Monolith NT.115 Pico instrument (NanoTemper Technologies) with 20% LED power and 40% MST power. The dissociation constant (*K*_D_) between BrpT and BTI was determined using the M.O. Affinity Analysis software v.2.3 (NanoTemper Technologies).

### In Vitro Transcription (IVT) Assay

IVT assays were conducted as described previously [29, 30] with some modifications. The 399-bp *brpN* upstream region containing the *brpN* promoter (P_*brpN*_) was amplified by PCR using the primers brpN_IVT_F and brpN_IVT_R (Table S1) and cloned into pRLG770, carrying *rrnB* terminator with EcoRI and HindIII [30, 31]. The resulting plasmid, pWW2301, was used as the template for the IVT reactions. 1.5 μg of the template DNA was incubated with 8 mM BrpT (or buffer as a control) and 1 mM BTI (or 1% DMSO) for 1 h at 25°C in a 34 μl reaction mixture containing RNA polymerase reaction buffer (New England BioLabs (NEB)). Then, the mixtures were supplemented with a nucleotide triphosphate (NTP) mixture (1 mM each NTP), 40 units of RNase inhibitor (Invitrogen, USA), and 1 unit of *E. coli* σ^70^-saturated RNA polymerase (Eσ^70^) (NEB). The reactions were incubated at 37°C for 2 h, followed by treatment with 1 unit of DNase I (Promega, USA) at 37°C for 30 min. The RNA transcript was purified using an RNeasy MinElute Cleanup kit (Qiagen), annealed with the 6FAM-labeled primer, PbrpN_IVT_6FAM (Table S1), and extended using SuperScript IV reverse transcriptase (Invitrogen) for 1 h at 50°C. For internal reference, a 399-bp 6FAM-labeled DNA was generated with unlabeled brpN_up_F and 6FAM-labeled brpN_up_R_6FAM (Table S1) and added to each sample to a final concentration of 0.01 ng/μl. The samples were analyzed using an ABI 3730xl DNA analyzer (Applied Biosystems) with Peak Scanner software v1.0 (Applied Biosystem). Relative levels of the transcripts were calculated by using the peak height of the 399-bp 6FAM-labeled DNA as a reference for normalization.

### Antibiotic Susceptibility Assay

*V. vulnificus* cultures grown to an *A*_600_ of 0.8 were diluted to an *A*_600_ of 0.05 in VFMG supplemented with 0.01%arabinose and 100 μM of BTI or 1% DMSO (control). The diluted culture (100 μl) was transferred to each well of a 96-well polystyrene microtiter plate (Nunc). After 24 h of incubation at 30°C, 100 μl of VFMG supplemented with 0.01% arabinose and various concentrations of ampicillin or 1% sterile distilled water (control) was added. After 8 h of incubation with shaking at 30°C, the biofilm was scraped from the microtiter well and resuspended in PBS. The bacterial suspension was serially diluted with PBS and appropriate dilutions were plated on LBS agar plates. The plates were incubated at 30°C for 24 h and the colony-forming units (CFU) were calculated. The viability of cells was shown in log_10_ reduction of CFU, calculated by dividing the CFU of ampicillin-treated samples by the CFU of non-ampicillin-treated control.

### Data Analysis

Data were analyzed using GraphPad Prism 9.0 (GraphPad Software) and presented as mean ± standard error of the mean (SEM) from at least three independent replicates. Statistical significance was determined by Student's *t*-test with a *p*-value < 0.05 considered significant.

## Results

### Discovery of Small-Molecule Inhibitors of BrpT Activity

**Fig. 1:**
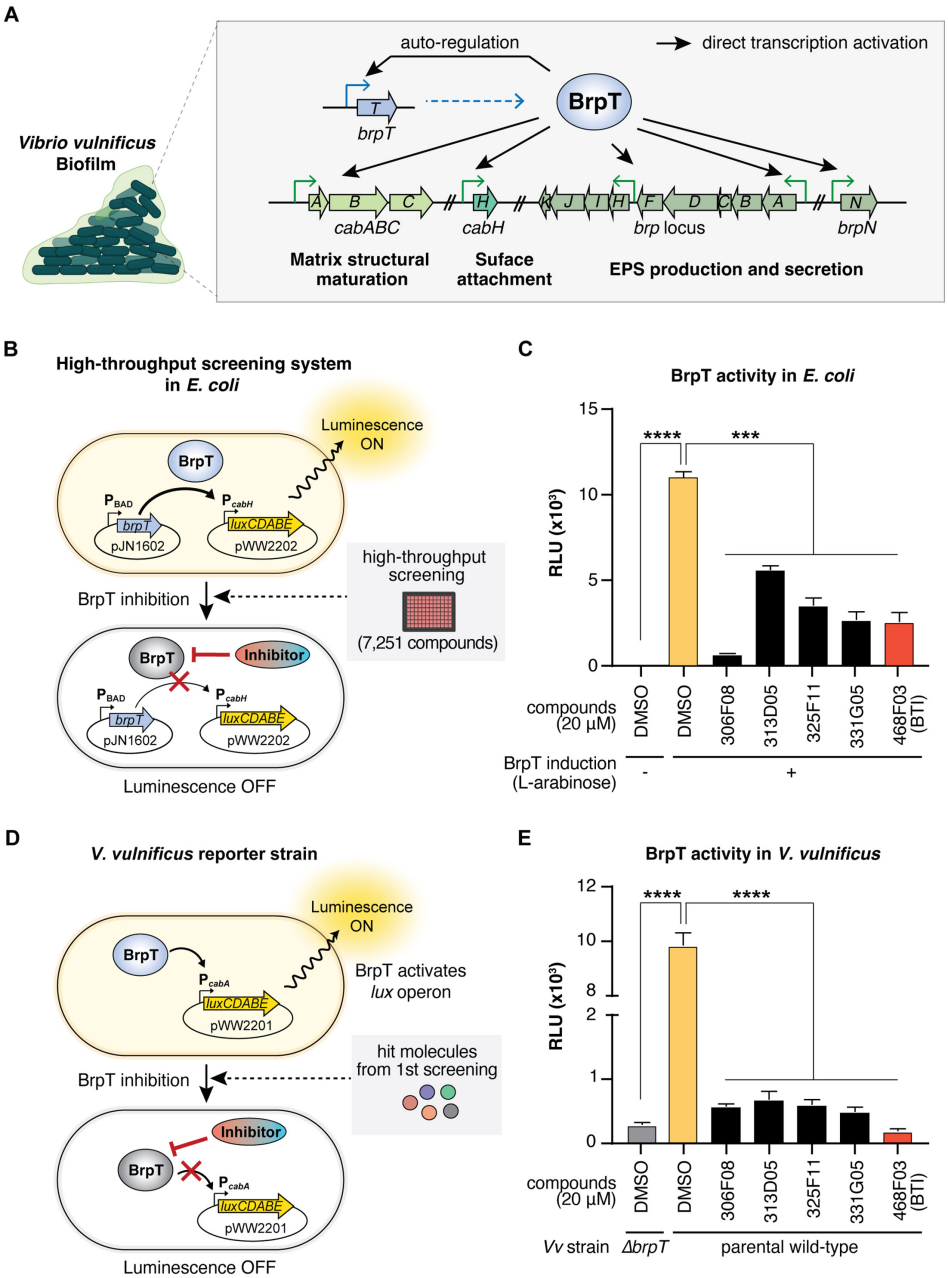
High-throughput screening for BrpT inhibitors. (**A**) Schematic representation of BrpT’s role in activating biofilm-related genes in *V. vulnificus*. (**B**) *E. coli*-based screening system. BrpT expression (pJN1602) was induced by 0.0002% L-arabinose. Active BrpT drives *lux* operon expression (pWW2202), producing luminescence. Inhibitors block this process are screened. (**C**) BrpT activity in *E. coli* measured by relative luminescence (**RLU**). Hit compounds significantly reduced BrpTinduced luminescence are shown. (**D**) *V. vulnificus* reporter strain for hit validation. Native BrpT activates P_*cabA*_ promoter (pWW2201), leading to *lux* operon expression. (**E**) BrpT activity in *V. vulnificus*. Hit compounds significantly reduced luminescence in a parental wild-type *V. vulnificus*. JN111, a parental wild-type strain, and *ΔbrpT*, a *brpT* deletion mutant, were used. Data represent means ± SEMs from three biological replicates. Statistical significance was determined by Student's *t*-test (****, *p* < 0.0001; ***, *p* < 0.0005). Vv, *V. vulnificus*.

We aimed to target BrpT to find selective and effective inhibitors of *V. vulnificus* biofilm formation (Fig. 1A). For high-throughput screening of BrpT inhibitors, we constructed the *E. coli* reporter strain containing pJN1602 (carrying the arabinose-inducible *brpT* of *V. vulnificus*) and pWW2202 (carrying the promoterless *lux* operon fused to the BrpT-activated promoter P_*cabH*_). Because the *cabH* gene is directly activated by BrpT [15], the *E. coli* reporter strain remains luminescent unless a potential hit molecule inhibits BrpT’s activity (Fig. 1B). This heterologous *E. coli* reporter system enabled us to exclude false-positive effects from molecules that reduce BrpT expression or target other *V. vulnificus* proteins contributing to *cabH* expression.

Due to the absence of a previously discovered ligand or a putative ligand-binding site in BrpT, we screened a random chemical library containing 7,251 compounds (at 20 μM) using the *E. coli* reporter strain. The screening identified five hit molecules (306F08, 313D05, 325F11, 331G05, and 468F03) (Table 2) that significantly reduced luminescence compared to the 2% DMSO control (Fig. 1C), indicating their ability to decrease the activity of the BrpT-induced promoter P_*cabH*_. The five hit molecules share common structural features that may contribute to their inhibitory activity against BrpT: multiple aromatic ring structures, including at least one nitrogen-containing heterocycle, short linkers connecting the ring systems, and a mix of polar and nonpolar regions (Table 2).

To validate the inhibitory effects of the hit molecules, we used *V. vulnificus* reporter strains: JN111 (parental wild-type) containing an inducible *dcpA* gene on its chromosome (P_BAD_-*dcpA*) to elevate c-di-GMP levels [14], and *ΔbrpT* (JN111 with a *brpT* deletion) as a negative control for BrpT-independent background luminescence. Both strains carried pWW2201 with the *lux* operon fused to the BrpT-activated promoter P_*cabA*_ (Fig. 1D), ensuring that inhibitory effects were specifically due to BrpT inhibition. Using a distinct promoter region in the *V. vulnificus* reporter strains minimized false positives from molecules binding to the *cabH* promoter in the *E. coli* screening system [32].

Consistent with the *E. coli* screening results, all five hit molecules significantly reduced luminescence in the *V. vulnificus* reporter strain, indicating effective inhibition of BrpT activity (Fig. 1E). Compound 468F03 showed the most potent inhibition and was selected for further analysis as a putative BrpT inhibitor.

### Identification of BTI as an Inhibitor of BrpT Activity

**Fig. 2:**
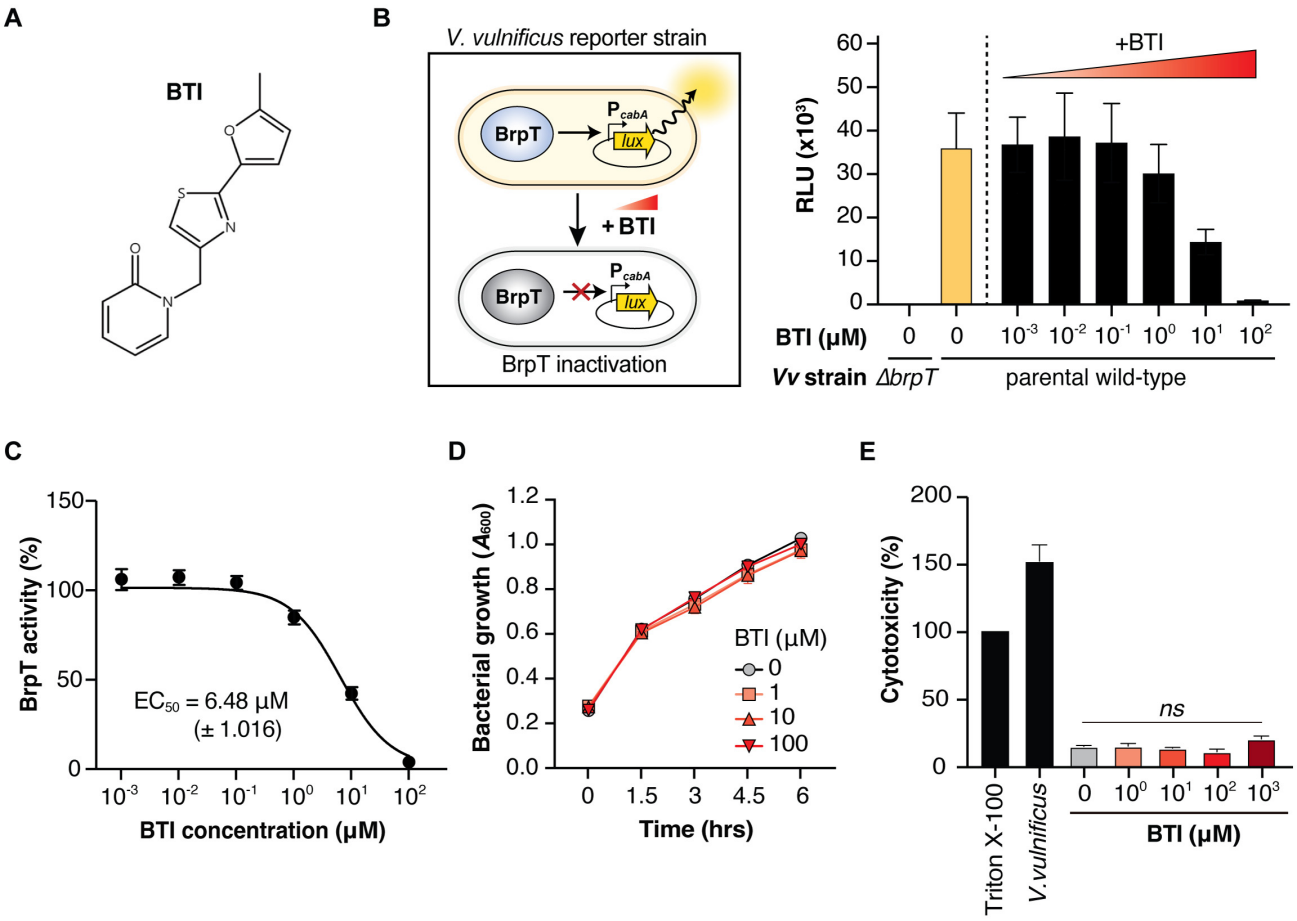
BTI inhibits BrpT activity without affecting *V. vulnificus* growth or cellular toxicity. (**A**) Chemical structure of BTI (1-{[2-(5-methylfuran-2-yl)-1,3-thiazol-4-yl]methyl}-1,2-dihydropyridin-2-one). (**B** and **C**) Dosedependent inhibition of BrpT activity by BTI in *V. vulnificus* reporter strains. (**B**) Schematic of *V. vulnificus* reporter system in which active BrpT induces luminescence via P_*cabA*_-controlled *lux* operon. RLU measurements at varying BTI concentrations are shown. (**C**) Quantification of BrpT activity. RLU of wild-type strain without BTI set as 100% and *ΔbrpT* as 0%. JN111, a parental wild-type strain, and *ΔbrpT*, a *brpT* deletion mutant, were used. (**D**) Growth of *V. vulnificus* wild-type strain in LBS in the presence of various BTI concentrations up to 100 μM. (**E**) Assessment of cytotoxicity of BTI towards human epithelial HeLa cells measured by released LDH activities after 3 h of incubation at 37°C. LDH activity from cells lysed with 3% Triton X-100 set as 100%. Cytotoxicity of the wild-type *V. vulnificus* at an MOI of 10 was shown for comparison. Data represent means ± SEMs from three independent experiments. Statistical significance was determined by Student’s *t*-test (ns, not significant). RLU, relative luminescence unit; MOI, multiplicity of infection; *Vv*, *V. vulnificus*.

The chemical structure of 468F03, 1-{[2-(5-methylfuran-2-yl)-1,3-thiazol-4-yl]methyl}-1,2-dihydropyridin-2-one (C_14_H_12_N_2_O_2_S), with a molecular weight of 272.3, was provided by the Korea Chemical Bank (Fig. 2A). Using a *V. vulnificus* reporter strain, we observed a dose-dependent decrease in BrpT activity with increasing concentrations of 468F03 (Fig. 2B), with the half-maximal effective concentration (EC_50_) of 6.48 μM (Fig. 2C). Since BrpT auto-regulates its own expression [11], the inhibitory effect of 468F03 is likely attributed to both a reduction in BrpT activity and a following decrease in BrpT expression.

Notably, 468F03 did not affect *V. vulnificus* growth (Fig. 2D), suggesting a lower potential for resistance development [33]. An LDH assay revealed no cytotoxicity in the human epithelial cell model, HeLa cells (Fig. 2E). Overall, these findings suggest that 468F03 is a small molecule that inhibits BrpT activity, with the potential to be developed as an anti-biofilm agent against *V. vulnificus*. We designated 468F03 as 'BTI' (BrpT Inhibitor) for further studies.

### BTI Diminishes the BrpT Regulon Expression and Biofilm Formation in *V. vulnificus*

**Fig. 3:**
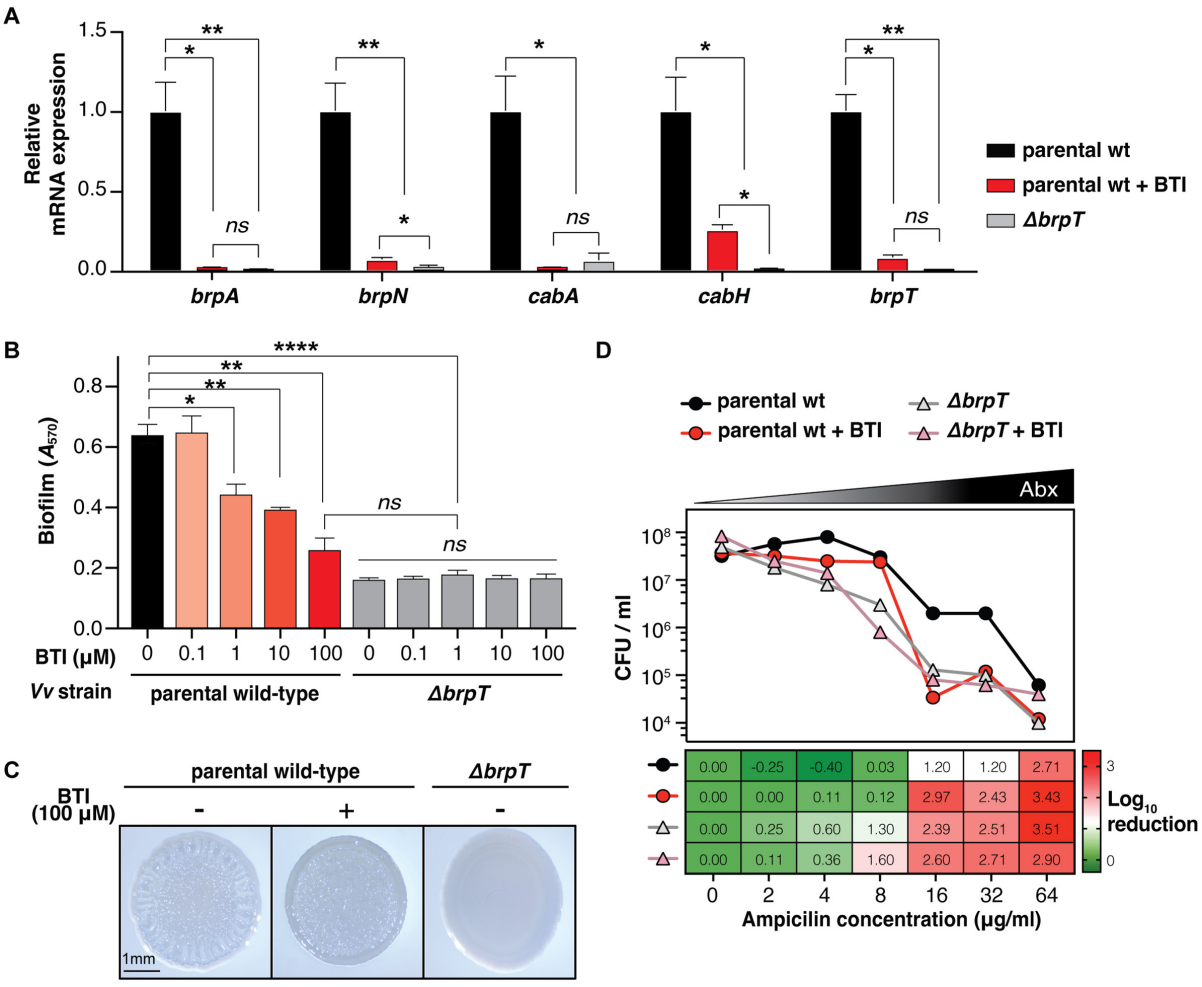
BTI reduces biofilm-related phenotypes of *V. vulnificus*. (**A**) Relative mRNA expression levels of *brpA*, *brpN*, *cabA*, *cabH*, and *brpT* relative to those in the parental wild-type (set as 1). (**B**) Quantification of biofilm formation in *V. vulnificus* strains grown with varying BTI concentrations. Biofilms were stained with crystal violet and measured at *A*_570_ after elution. (**C**) Representative image of colony rugosity of *V. vulnificus* strains in the presence or absence of 100 μM BTI after 24 h incubation. Scale bar represents 1 mm. (**D**) Survival of *V. vulnificus* against antibiotics. Biofilms were grown with 100 μM BTI (or 1% DMSO for control) for 24 h and then treated with various concentrations of ampicillin for 8 h. Viable cells after ampicillin treatment were counted. JN111, a parental wild-type strain, and *ΔbrpT*, a *brpT* deletion mutant, were used. Data represent means ± SEMs from three independent experiments. Statistical significance was determined by Student’s *t*-test (****, *p* < 0.0001; ***, *p* < 0.0005; **, *p* < 0.005; *, *p* < 0.05; ns, not significant). wt, wild-type; Vv, *V. vulnificus*.

Because BrpT is a crucial transcription regulator for multiple biofilm-associated genes (Fig. 1A), we investigated BTI's effect on the BrpT regulon. BTI (100 μM) significantly diminished the expression of BrpT-induced genes (*brpA*, *brpN*, *cabA*, *cabH*, and *brpT*) in the parental wild-type to the levels comparable to those in the *ΔbrpT* (Fig. 3A), consistent with its inhibition of BrpT activity (Fig. 2C). Additionally, BTI did not alter the expression of genes not regulated by BrpT, such as *rtxA*, *hlyU*, and *smcR*, indicating its specificity for BrpT-regulated genes (Fig. S1).

We then assessed the impact of BTI on *V. vulnificus* biofilm formation using crystal violet staining. *ΔbrpT* exhibited markedly impaired biofilm development as demonstrated previously [17], confirming the essential role of BrpT in biofilm formation. BTI treatment caused a significant, dose-dependent reduction of biofilm formation in the parental wild-type strain, but not in *ΔbrpT* (Fig. 3B), confirming its selective effect on BrpT.

Furthermore, to examine the effect of BTI on colony rugosity, the parental wild-type and the *ΔbrpT* strains were grown on VFMG agar supplemented with 0.02% arabinose and 100 μM BTI (or 0.1% DMSO for control) for 24 h. While the parental wild-type treated with 0.1% DMSO displayed pronounced rugose colony morphology, treatment with 100 μM of BTI resulted in significantly smoother colonies, comparable to those of *ΔbrpT* (Fig. 3C).

To explore the impact of impaired *V. vulnificus* biofilm formation by BTI on bacterial susceptibility to antibiotics, the parental wild-type and the *ΔbrpT* strains, grown on VFMG supplemented with 0.01% arabinose and 100 μM BTI (or 1% DMSO for control) for 24 h, were treated with various concentrations of ampicillin (0 -64 μg/ml) and then viable cells were counted. The parental wild-type strain treated with 1% DMSO showed notable reduction in viable cell counts only at 64 μg/ml ampicillin (2.71 log_10_ reduction). In contrast, both the BTI-treated wild-type strain and *ΔbrpT* (with or without BTI) exhibited increased sensitivity, with substantial reductions (>2 log_10_) starting at 16 μg/ml ampicillin (Fig. 3D). These findings collectively demonstrate that BTI effectively inhibits BrpT-dependent biofilm development in *V. vulnificus*, thereby enhancing antibiotic sensitivity. This highlights BTI’s potential as an anti-biofilm agent to improve antibiotics efficacy against *V. vulnificus* infection.

### BTI Directly Binds to BrpT and Inhibits Its Transcriptional Activity In Vitro

**Fig. 4:**
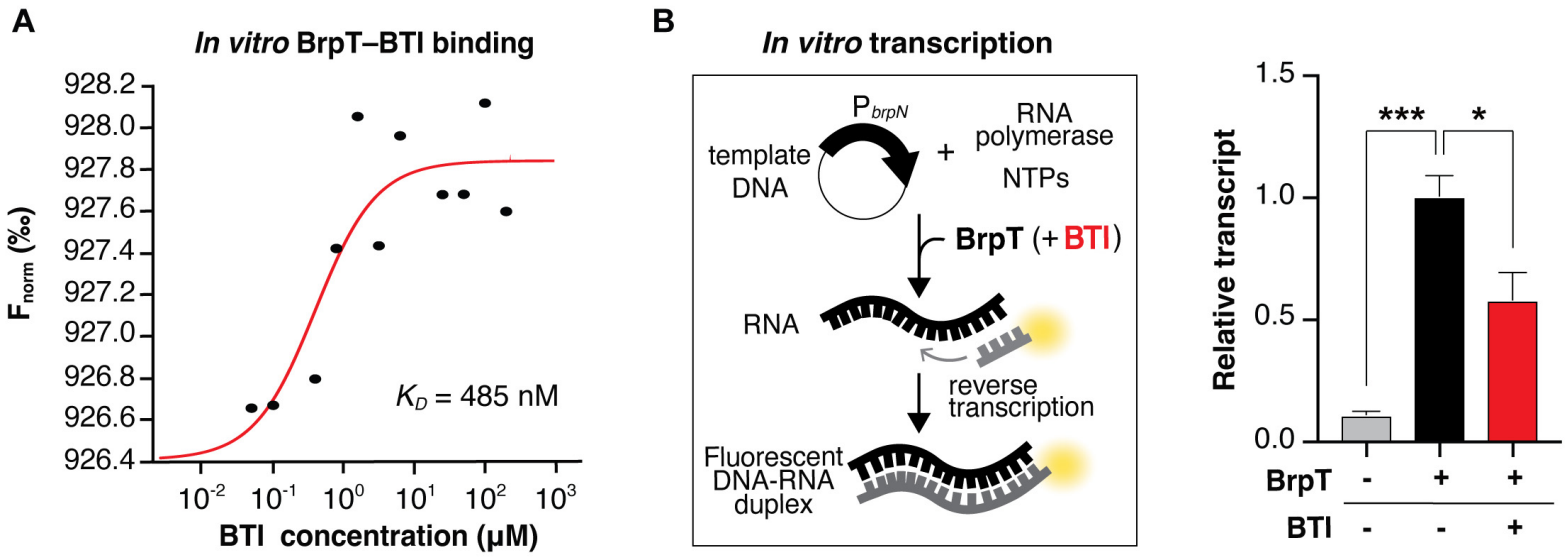
BTI directly binds to BrpT and attenuates transcription in vitro. (**A**) Microscale thermophoresis analysis demonstrating the direct interaction between BTI and BrpT. The normalized thermophoresis values of BrpT (F_norm_) at varying BTI concentrations are shown. (**B**) In vitro transcription assay determining the effect of BTI on BrpT-mediated transcriptional activation of the *brpN* promoter. A template DNA containing P_*brpN*_ was transcribed in vitro with 8 μM BrpT in the presence or absence of 1 mM BTI as indicated. Relative levels of the transcripts were determined using the heights of the transcript peaks measured in arbitrary fluorescent units, shown with that of BrpT without BTI set as 1. Error bars represent the SEM from three independent experiments. Statistical significance was determined by Student’s *t*-test (***, *p* < 0.0005; *, *p* < 0.05).

To elucidate the mechanism by which BTI inhibits BrpT’s activity as a transcription regulator, we conducted in vitro assays using purified BrpT protein. First, the direct interaction between BTI and BrpT was investigated using MST, wherein fluorescently labeled BrpT proteins were reacted with varying BTI concentrations. The result clearly indicates the direct binding of BTI to BrpT, as detected by the shifts in fluorescence intensity representing the changes in the thermophoretic movement of BrpT caused by BTI binding (Fig. 4A).

Next, the effect of BTI on BrpT activity as a transcription factor was analyzed using an IVT assay. The promoter region of *brpN*, P_*brpN*_, was cloned into pRLG770 vector and used as a DNA template for transcription assay. The presence of BrpT in the reaction enabled the transcription of P_*brpN*_, which is negligible in its absence, clearly indicating BrpT’s role as a transcriptional activator of *brpN*. Importantly, BTI treatment significantly reduced the transcription level of *brpN* in the presence of BrpT (Fig. 4B), indicating that BTI impedes the activity of BrpT in inducing transcription. Taken together, these results demonstrate that BTI directly binds to BrpT and inhibits its transcriptional regulatory activity.

## Discussion

In this study, we identified BTI, a small molecule inhibitor that effectively inhibits the activity of BrpT, a key transcriptional regulator of biofilm formation in the human pathogen *V. vulnificus* (Figs. 1 and 2). We demonstrated that BTI specifically binds to BrpT and diminishes its transcriptional regulatory activity (Fig. 4), leading to decreased expression of essential biofilm genes, impaired biofilm formation, and increased antibiotic sensitivity (Fig. 3). The autoregulatory positive feedback loop of BrpT [11] amplifies the effects of BTI, enabling effective biofilm inhibition at lower drug concentrations, which could enhance its potential safety for therapeutic use.

The alarming rise of antibiotic resistance has necessitated the development of alternative strategies to combat bacterial infections. Targeting virulence and biofilm formation presents a promising approach to mitigate infection without exerting direct selective pressure for resistance [6, 18]. BTI exemplifies this strategy as the first small molecule inhibitor to directly target BrpT, a master transcriptional regulator of biofilm formation in *V. vulnificus*. This unique mechanism of action distinguishes BTI from previously reported inhibitors that primarily target quorum sensing [34], utilize unidentified natural products [35], or inhibit bacterial growth [36]. By specifically inhibiting BrpT, BTI affects the expression of a wide range of biofilm-related genes without impacting bacterial growth (Fig. 2). This targeted approach to biofilm inhibition could potentially reduce *V. vulnificus* colonization and virulence in both environmental and host niches [37, 38], while minimizing the risk of resistance development. This offers a significant advantage over conventional antibiotics [6]. Future studies could explore the potential synergy between BTI and existing biofilm inhibitors or antibiotics to enhance overall efficacy against biofilm-associated infections, particularly in light of the increased antibiotic resistance often conferred by biofilms (Fig. 3) [39].

Another important area for future study is the potential broad-spectrum activity of BTI against other *Vibrio* species. Amino acid sequence analyses suggest that BrpT and its homologs in other *Vibrio* species share conserved structural features, including the C-terminal DNA-binding domain and the c-di-GMP binding region (Fig. S2)[40]. This raises the tantalizing possibility that BTI-based therapies could be effective against biofilm formation by other clinically relevant *Vibrio* pathogens, such as *V. cholerae* and *V. parahaemolyticus*, because biofilm formation is a critical factor in the pathogenesis of these species [41, 42]. Future studies elucidating the detailed molecular interactions between BTI and BrpT, as well as the conservation of the BTI binding site across BrpT homologs, will be crucial to explore the broader therapeutic potential of this inhibitor.

As a transcriptional regulator, BrpT directly binds to the promoter region of its regulon and facilitates the recruitment of RNA polymerase to promote transcription [32, 43]. While the detailed molecular mechanism of BTI-mediated inhibition requires further investigation, two potential inhibitory modes can be proposed; BTI may reduce BrpT’s affinity for target promoters by inducing a conformational change in BrpT [32, 44]. Alternatively, BTI may impede BrpT-RNA polymerase interactions, hindering RNA polymerase binding to the promoter region [32, 44, 45]. Both modes effectively block BrpT-mediated biofilm gene regulation in *V. vulnificus* [4, 6], and future structural and biochemical studies will illuminate the precise molecular mechanism.

In conclusion, our study identified BTI as a promising small-molecule inhibitor of BrpT that effectively inhibits *V. vulnificus* biofilm formation. By targeting a master regulator of biofilm development, BTI exemplifies a novel strategy to combat *V. vulnificus* infections while potentially minimizing the risk of resistance. Our findings lay the foundation for future studies to optimize the structure and efficacy of BTI, investigate its broader spectrum of activity against *Vibrio* biofilms, and advance its therapeutic development. The discovery of BTI represents a significant step forward in developing alternative anti-infective therapies, which are urgently needed to combat the rising threat of antibiotic resistance.

## Supplemental Materials

Supplementary data for this paper are available on-line only at http://jmb.or.kr.



## Figures and Tables

**Table 1:** Bacterial strains and plasmids used in this study.

Strain or plasmid	Relevant characteristics ^a^	Reference of source
*V. vulnificus*		
CMCP6	Wild type; Clinical isolate; virulent	Laboratory collection
JN111	CMCP6 with P_BAD_-*dcpA*	[14]
JN161D	JN111 with *ΔbrpT*	[46]
*E. coli*		
DH5α	*supE44 ΔlacU169* (*Φ80 lacZ Δ*M15) *hsdR17 recA1 endA1 gyrA96 thi-1 relAI*	Laboratory collection
S17-1 λpir	λ-*pir* lysogen; *thi pro hsdR hsdM*^0+^ *recA* RP4-2 Tc::Mu-Km::Tn7;Tp^r^ Smr^;^ host for π-requiring plasmids	[47]
BL21 (DE3)	F^-^, *ompT*, *hsdS* (r_B_^-^, m_B_^-^), *gal dcm* (DE3)	Laboratory collection
Plasmids		
pBBR-lux	Broad host range vector with promoterless *luxCDABE*; Cm^r^	[48]
pJN1602	pJK1113 with *brpT*; Ap^r^ Km^r^	[17]
pWW2201	pBBR-lux with 326-bp fragment of *cabA* upstream region; Cm^r^	This study
pWW2202	pBBR-lux with 336-bp fragment of *cabH* upstream region; Cm^r^	This study
pSH1819	pET-28a(+) with *brpT*; Km^r^	[17]
pRLG770	General transcription vector; Ap^r^	[49]
pWW2301	pRLG770 with 399-bp fragment of *brpN* upstream region;Ap^r^	This study

^a^Tp^r^, trimethoprim resistant; Sm^r^, streptomycin resistant; Cm^r^, chloramphenicol resistant; Ap^r^, ampicillin resistant; Km^r^, kanamycin resistant.

**Table 2:** The structure of hit molecules identified from the high-throughput screening.

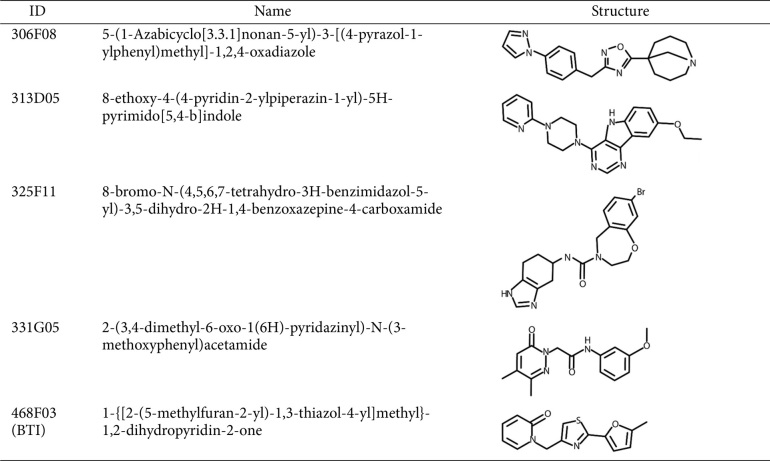

## References

[1] Coerdt KM, Khachemoune A (2021). *Vibrio vulnificus*: review of mild to life-threatening skin infections. Cutis..

[2] Baker-Austin C, Oliver JD (2018). *Vibrio vulnificus*: new insights into a deadly opportunistic pathogen. Environ. Microbiol..

[3] Baker-Austin C, Oliver JD, Alam M, Ali A, Waldor MK, Qadri F (2018). *Vibrio* spp. infections. Nat. Rev. Dis. Primers.

[4] Rippey SR (1994). Infectious diseases associated with molluscan shellfish consumption. Clin. Microbiol. Rev..

[5] Kim SM, Park JH, Lee HS, Kim WB, Ryu JM, Han HJ (2013). LuxR Homologue SmcR is essential for pathogenesis and biofilm detachment, and its expression is induced by host cells. Infect. Immun..

[6] Cegelski L, Marshall GR, Eldridge GR, Hultgren SJ (2008). The biology and future prospects of antivirulence therapies. Nat. Rev. Microbiol..

[7] Hall-Stoodley L, Costerton JW, Stoodley P (2004). Bacterial biofilms: from the natural environment to infectious diseases. Nat. Rev. Microbiol..

[8] Hengzhuang W, Wu H, Ciofu O, Song Z, Hoiby N (2011). Pharmacokinetics/pharmacodynamics of colistin and imipenem on mucoid and nonmucoid *Pseudomonas aeruginosa* biofilms. Antimicrob. Agents Chemother..

[9] Mah TF (2012). Biofilm-specific antibiotic resistance. Future Microbiol..

[10] Hall-Stoodley L, Stoodley P (2005). Biofilm formation and dispersal and the transmission of human pathogens. Trends Microbiol..

[11] Chodur DM, Rowe-Magnus DA (2018). Complex control of a genomic island governing biofilm and rugose colony development in *Vibrio vulnificus*. J. Bacteriol..

[12] Guo Y, Rowe-Magnus DA (2010). Identification of a c-di-GMP-regulated polysaccharide locus governing stress resistance and biofilm and rugose colony formation in *Vibrio vulnificus*. Infect. Immun..

[13] Garrison-Schilling KL, Kaluskar ZM, Lambert B, Pettis GS (2014). Genetic analysis and prevalence studies of the *brp* exopolysaccharide locus of *Vibrio vulnificus*. PLoS One.

[14] Park JH, Jo Y, Jang SY, Kwon H, Irie Y, Parsek MR (2015). The *cabABC* operon essential for biofilm and rugose colony development in *Vibrio vulnificus*. PLoS Pathog..

[15] Lee H, Im H, Hwang SH, Ko D, Choi SH (2023). Two novel genes identified by large-scale transcriptomic analysis are essential for biofilm and rugose colony development of *Vibrio vulnificus*. PLoS Pathog..

[16] Chodur DM, Coulter P, Isaacs J, Pu M, Fernandez N, Waters CM (2018). Environmental calcium initiates a feed-forward signaling circuit that regulates biofilm formation and rugosity in *Vibrio vulnificus*. mBio.

[17] Hwang SH, Park JH, Lee B, Choi SH (2020). A regulatory network controls *cabABC* expression leading to biofilm and rugose colony development in *Vibrio vulnificus*. Front. Microbiol..

[18] Ghosh A, Jayaraman N, Chatterji D (2020). Small-molecule inhibition of bacterial biofilm. ACS Omega.

[19] Parrino B, Schillaci D, Carnevale I, Giovannetti E, Diana P, Cirrincione G (2019). Synthetic small molecules as anti-biofilm agents in the struggle against antibiotic resistance. Eur. J. Med. Chem..

[20] Cao X, Studer SV, Wassarman K, Zhang Y, Ruby EG, Miyashiro T (2012). The novel sigma factor-like regulator RpoQ controls luminescence, chitinase activity, and motility in *Vibrio fischeri*. mBio.

[21] Nakhamchik A, Wilde C, Rowe-Magnus DA (2008). Cyclic-di-GMP regulates extracellular polysaccharide production, biofilm formation, and rugose colony development by *Vibrio vulnificus*. Appl. Environ. Microbiol..

[22] Guzman LM, Belin D, Carson MJ, Beckwith J (1995). Tight regulation, modulation, and high-level expression by vectors containing the arabinose P_BAD_ promoter. J. Bacteriol..

[23] Masters JR (2002). HeLa cells 50 years on: the good, the bad and the ugly. Nat. Rev. Cancer.

[24] Ko D, Sung D, Kim TY, Choi G, Bang YJ, Choi SH (2023). CarRS two-component system essential for polymyxin B resistance of *Vibrio vulnificus* responds to multiple host environmental signals. Microbiol. Spectr..

[25] Lim JG, Choi SH (2014). IscR is a global regulator essential for pathogenesis of *Vibrio vulnificus* and induced by host cells. Infect. Immun..

[26] Williams TC, Blackman ER, Morrison SS, Gibas CJ, Oliver JD (2014). Transcriptome sequencing reveals the virulence and environmental genetic programs of *Vibrio vulnificus* exposed to host and estuarine conditions. PLoS One.

[27] Ko D, Choi SH (2021). Comparative genomics reveals an SNP potentially leading to phenotypic diversity of *Salmonella enterica* serovar Enteritidis. Microb. Genom..

[28] Lim MS, Kim JA, Lim JG, Kim BS, Jeong KC, Lee KH, Choi SH (2011). Identification and characterization of a novel serine protease, VvpS, that contains two functional domains and is essential for autolysis of *Vibrio vulnificus*. J. Bacteriol..

[29] Lee ZW, Hwang SH, Choi G, Jang KK, Lee TH, Chung KM (2020). A MARTX Toxin rtxA gene is controlled by host environmental signals through a CRP-coordinated regulatory network in *Vibrio vulnificus*. mBio.

[30] Kim BS, Hwang J, Kim MH, Choi SH (2011). Cooperative regulation of the *Vibrio vulnificus* nan gene cluster by NanR protein, cAMP receptor protein, and N-acetylmannosamine 6-phosphate. J. Biol. Chem..

[31] Sudzinova P, Kambova M, Ramaniuk O, Benda M, Sanderova H, Krasny L (2021). Effects of DNA topology on transcription from rRNA promoters in *Bacillus subtilis*. Microorganisms.

[32] Berg T (2008). Inhibition of transcription factors with small organic molecules. Curr. Opin. Chem. Biol..

[33] Pontes MH, Groisman EA (2019). Slow growth determines nonheritable antibiotic resistance in *Salmonella enterica*. Sci. Signal..

[34] Packiavathy IA, Sasikumar PS, Pandian SK, VeeraRavi A (2013). Prevention of quorum-sensing-mediated biofilm development and virulence factors production in *Vibrio* spp. by curcumin. Appl. Microbiol. Biotechnol..

[35] Ohn HM, Mizuno T, Sudo Y, Miyoshi SI (2020). Interaction of *Escherichia coli* and its culture supernatant with *Vibrio vulnificus* during biofilm formation. Microbiol. Immunol..

[36] Luo KY, Kang SN, Guo M, Shen CY, Wang LH, Xia XD (2022). Evaluation of the antibacterial mechanism and biofilm removal effect of eugenol on *Vibrio vulnificus* and its application in fresh oysters. Food Biosci..

[37] Bross MH, Soch K, Morales R, Mitchell RB (2007). *Vibrio vulnificus* infection: diagnosis and treatment. Am. Fam. Phys..

[38] Choi G, Choi SH (2022). Complex regulatory networks of virulence factors in *Vibrio vulnificus*. Trends Microbiol..

[39] Arunkumar M, LewisOscar F, Thajuddin N, Pugazhendhi A, Nithya C (2020). and biofilm forming spp: a significant threat in aquaculture. Process Biochem..

[40] Krasteva PV, Fong JC, Shikuma NJ, Beyhan S, Navarro MV, Yildiz FH (2010). *Vibrio cholerae* VpsT regulates matrix production and motility by directly sensing cyclic di-GMP. Science.

[41] Fong JCN, Syed KA, Klose KE, Yildiz FH (2010). Role of *Vibrio* polysaccharide (vps) genes in VPS production, biofilm formation and *Vibrio cholerae* pathogenesis. Microbiology (Reading).

[42] Wang J, Zhan Y, Sun H, Fu X, Kong Q, Zhu C, Mou H (2022). Regulation of Virulence Factors Expression During the Intestinal Colonization of *Vibrio parahaemolyticus*. Foodborne Pathog Dis..

[43] Popham DL, Szeto D, Keener J, Kustu S (1989). Function of a bacterial activator protein that binds to transcriptional enhancers. Science.

[44] Mobley DL, Dill KA (2009). Binding of small-molecule ligands to proteins: "what you see" is not always "what you get". Structure.

[45] Buchwald P (2010). Small-molecule protein-protein interaction inhibitors: therapeutic potential in light of molecular size, chemical space, and ligand binding efficiency considerations. IUBMB Life.

[46] Hwang SH, Im H, Choi SH (2021). A master regulator BrpR coordinates the expression of multiple loci for robust biofilm and rugose colony development in *Vibrio vulnificus*. Front. Microbiol..

[47] Simon R, Priefer U, Puhler A (1983). A broad host range mobilization system for in vivo genetic-engineering - Transposon mutagenesis in gram-negative bacteria. Bio-Technol..

[48] Lenz DH, Mok KC, Lilley BN, Kulkarni RV, Wingreen NS, Bassler BL (2004). The small RNA chaperone Hfq and multiple small RNAs control quorum sensing in *Vibrio harveyi* and *Vibrio cholerae*. Cell.

[49] Ross W, Thompson JF, Newlands JT, Gourse RL (1990). *E. coli* Fis protein activates ribosomal RNA transcription in vitro and in vivo. EMBO J..

