# Supplementary material for: Discovery of a Small-Molecule Inhibitor Targeting the Biofilm Regulator BrpT in Vibrio vulnificus
Source: J Microbiol Biotechnol. 2024 Sep 20;34(11):2201–10. doi: 10.4014/jmb.2406.06052 (PMC11637837; doi:10.4014/jmb.2406.06052)
Supplement: Supplementary file 1 [file jmb-34-11-2201-supple.pdf]

J. Microbiol. Biotechnol. 2024. 34(11): 2201–2210

https://doi.org/10.4014/jmb.2406.06052

Supplementary Table and Figures

Discovery of a Small-Molecule Inhibitor Targeting the Biofilm Regulator BrpT in *Vibrio vulnificus*

Wonwoo Choi, Hojun Lee, Qiyao Wang, Ye-Ji Bang and Sang Ho Choi

Table S1. Oligonucleotides used in this study.

Oligonucleotide	Oligonucleotide sequence, 5' →3'[a]	Use
For reporter strain construction		
PcabA-lux_F	ATGAGCTCAAACCGCCTGGCCACTATTT	Amplification of *cabA* upstream region for reporter strain construction
PcabA-lux_R	TAGGATCCGCAGTTCCAGAATAAACAGCCA	
PcabH-lux_F	ATGAGCTCTAGTCTTACCAAGCTAGACCCGT	Amplification of *cabH* upstream region for reporter strain construction
PcabH-lux_R	TAGGATCCCAACCGTTGTTCCAGACATAGAT	
For *in vitro* transcription		
brpN_up_F	ATGAATTCGCATCAGTCTAAACACCGCAC	Amplification of *brpN* upstream region for IVT strain construction
brpN_up_R	TAAAGCTTAGGCAGTACAACCGCTGAGA	
brpN_up_R_6FAM	AGGCAGTACAACCGCTGAGA	Amplification of *brpN* upstream region for IVT reference
PbrpN_IVT_6FAM	GCGTATACATGAACCATAACCACCA	*in vitro* transcription of *brpN* upstream region.
For qRT-PCR		
brpA_qRT_F	CATCGGCTTTATGGCCTTGC	Quantification of *brpA* expression
brpA_qRT_R	GCGCTTTCGGCAAAGAGAAT	
brpN_qRT_F	GCTCAACCGCAGATTATGGA	Quantification of *brpN* expression
brpN_qRT_R	ATGTAGTGGAGCGCAAAGGA	
cabA_qRT__F	TTGGTTGCTGGCTCTGGTGAC	Quantification of *cabA* expression
cabA_qRT__R	ACTGTCTATACGCACTGTGTCCTC	
cabH_qRT_F	ATAAGTTCGTCGGCTTCGGG	Quantification of *cabH* expression
cabH_qRT_R	CCGCCAAAGAGGGTGTCATT	
brpT_qRT_F	GAAGCTGTGTCGCGGGATTG	Quantification of *brpT* expression
brpT_qRT_R	TGTGGCTCTTCCTTCTTCGCTC	

[a] Regions of oligonucleotides not complementary to the corresponding genes are underlined.

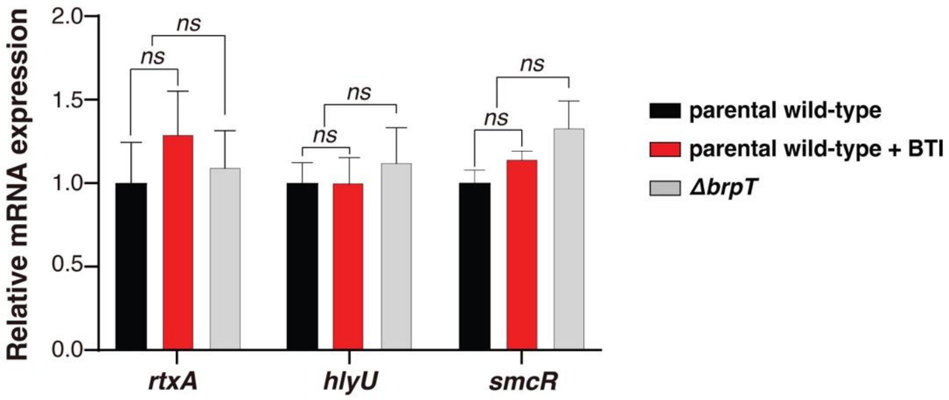

Fig. S1. BTI does not affect the expression of the non-BrpT regulon. Relative mRNA expression levels of *rtxA, hlyU,* and *smcR* as relative to those in the parental wild-type. JN111, a parental wild-type strain, and *ΔbrpT*, a *brpT* deletion mutant, were used. Data represent means ± SEMs from three independent experiments. Statistical significance was determined by Student's *t*-test (*ns*, not significant).

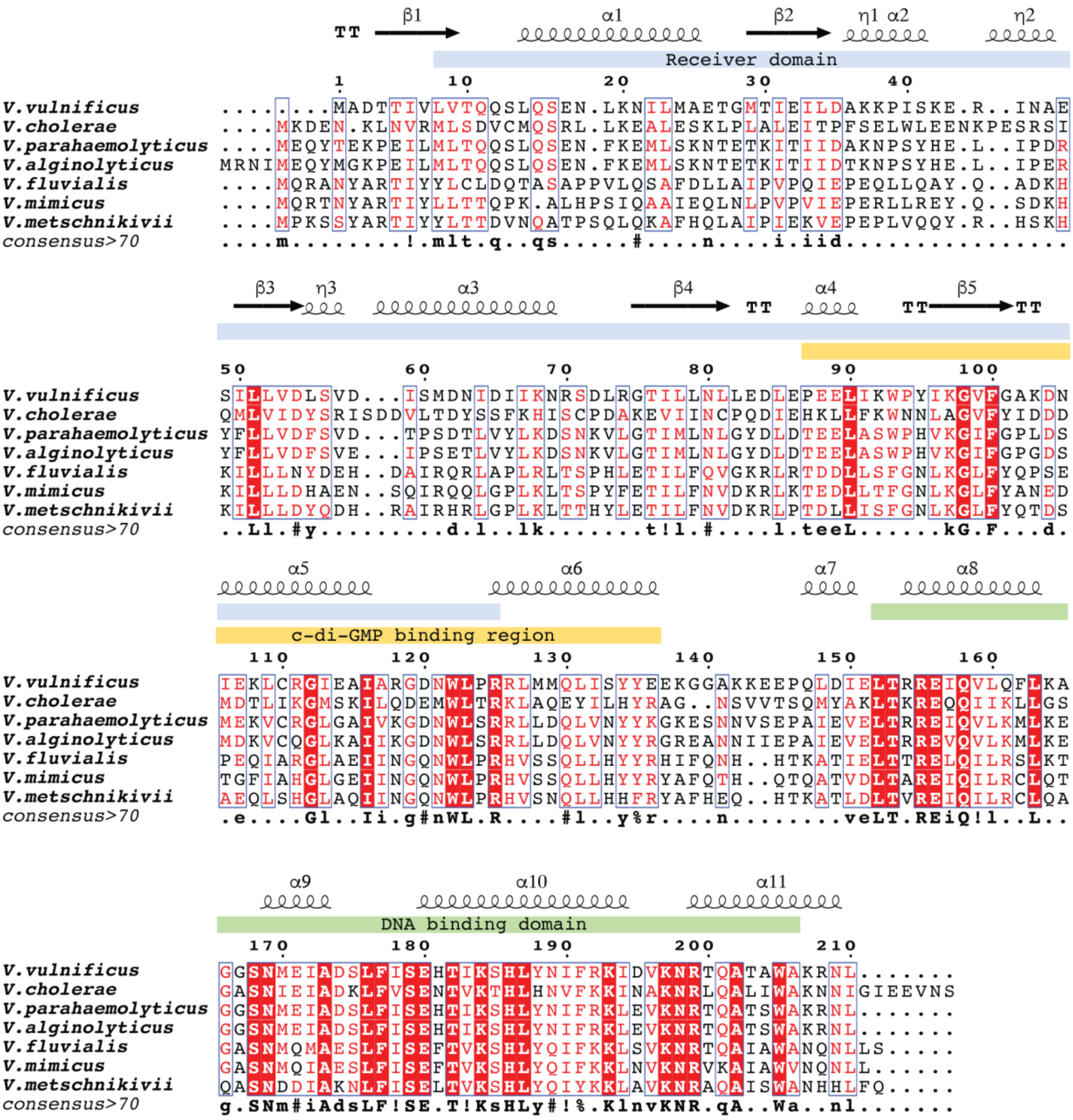

TT β1 α1 β2 η1 α2 η2
Receiver domain
1 10 20 30 40
V.vulnificus ........MADTTIVLVTQQSLQSEN.LKNILMAETGMTIEILDAKKPISKE.R..INAE
V.cholerae ....MKDEN.KLNVRMLSDVCMQSRL.LKEALESKLPLALEITPFSELWLEENKPESRSI
V.parahaemolyticus ....MEQYTEKPEILMLTQQSLQSEN.FKEMLSKNTETKITIIDAKNPSYHE.L..IPDR
V.alginolyticus MRNIMEQYMGKPEILMLTQQSLQSEN.FKEMLSKNTETKITIIDTKNPSYHE.L..IPER
V.fluvialis ....MQRANYARTIYYLCLDQTASAPPVLQSAFDLLAIPVPQIEPEQLLQAY.Q..ADKH
V.mimicus ....MQRTNYARTIYLLTTQPK.ALHPSIQAAIEQLNLPVPVIEPERLLREY.Q..SDKH
V.metschnikivii ....MPKSSYARTIYYLTTDVNQATPSQLQKAFHQLAIPIEKVEPEPLVQQY.R..HSKH
consensus>70 ....m........!.mlt.q..qs.....#....n....i.iid...............
β3 η3 α3 β4 TT α4 TT β5 TT
50 60 70 80 90 100
V.vulnificus SILLVDLSVD...ISMDNIDIIKNRSDLRGTILLNLLEDLEPEELIKWPYIKGVFGAKDN
V.cholerae QMLVIDYSRISDDVLTDYSSFKHISCPDAKEVIINCPQDIEHKLLFKWNNLAGVFYIDDD
V.parahaemolyticus YFLLVDFSVD...TPSDTLVYLKDSNKVLGTIMLNLGYDLDTEELASWPHVKGIFGPLDS
V.alginolyticus YFLLVDFSVE...IPSETLVYLKDSNKVLGTIMLNLGYDLDTEELASWPHVKGIFGPGDS
V.fluvialis KILLLNYDEH..DAIRQRLAPLRLTSPHLETILFQVGKRLRTDDLLSFGNLKGLFYQPSE
V.mimicus KILLLDHAEN..SQIRQQLGPLKLTSPYFETILFNVDKRLKTEDLLTFGNLKGLFYANED
V.metschnikivii KILLLDYQDH..RAIRHRLGPLKLTTHYLETILFNVDKRLPTDLLISFGNLKGLFYQTDS
consensus>70 ..Ll.#y.........d.l..lk.......t!l.#....l.teeL......kG.F...d.
α5 α6 α7 α8
c-di-GMP binding region
110 120 130 140 150 160
V.vulnificus IEKLCRGIEAIARGDNWLPRRLMMQLISYYEEKGGAKKEEPQLDIELTRREIQVLQFLKA
V.cholerae MDTLIKGMSKILQDEMWLTRKLAQEYILHYRAG..NSVVTSQMYAKLTKREQQIIKLLGS
V.parahaemolyticus MEKVCRGLGAIVKGDNWLSRRLLDQLVNYYKGKESNNVSEPAIEVELTRREIQVLKMLKE
V.alginolyticus MDKVCQGLKAIIKGDNWLSRRLLDQLVNYYRGREANNIIEPAIEVELTRREVQVLKMLKE
V.fluvialis PEQIARGLAEIINGQNWLPRHVSSQLLHYYRHIFQNH..HTKATIELTTRELQILRSLKT
V.mimicus TGFIAHGLGEIINGQNWLPRHVSSQLLHYYRYAFQTH..QTQATVDLTARELQILRCLQT
V.metschnikivii AEQLSHGLAQIINGQNWLPRHVSNQLLHHFRYAFHEQ..HTKATLDLTVREIQILRCLQA
consensus>70 .e....Gl..Ii.g#nWL.R....#l..y%r....n........veLT.REiQ!l..L..
α9 α10 α11
DNA binding domain
170 180 190 200 210
V.vulnificus GGSNMEIADSLFISEHTIKSHLYNIFRKIDVKNRTQATAWAKRNL.......
V.cholerae GASNIEIADKLFVSENTVKTHLHNVFKKINAKNRLQALIWAKNNIGIEEVNS
V.parahaemolyticus GGSNMEIADSLFISEHTIKSHLYNIFRKLEVKNRTQATSWAKRNL.......
V.alginolyticus GGSNMEIADSLFISEHTIKSHLYNIFRKLEVKNRTQATSWAKRNL.......
V.fluvialis GASNMQMAESLFISEFTVKSHLYQIFKKLSVKNRTQAIAWANQNLLS.....
V.mimicus GASNMQIAESLFISEFTVKSHLYQIFKKLNVKNRVKAIAWVNQNLL......
V.metschnikivii QASNDDIAKNLFISELTVKSHLYQIYKKLAVKNRAQAISWANHHLFQ.....
consensus>70 g.SNm#iAdsLF!SE.T!KsHLy#!%.KlnvKNR.qA..Wa..nl.......

Fig. S2. Conservation of BrpT homologs in pathogenic *Vibrio* species. Amino acid sequence alignment of BrpT homologs from representative pathogenic *Vibrio* species [1]. The alignment was generated with Clustal Omega [2] and ESPript 3.0 [3]. Secondary structure and domains are shown based on the crystal structure of *V. cholerae* VpsT (PDB:3KLN) [4]. The N-terminal receiver domain and the C-terminal helix-turn-helix DNA binding domain are indicated with blue and green bars, respectively. The region forming a dimer interface and a c-di-GMP binding pocket in VpsT is shown with a yellow bar. Residues that are strictly conserved across all sequences are highlighted in red, while similar residues are shown in red text. The following protein sequences were used to generate the alignment: AAO08433.1 (*V. vulnificus* BrpT), AAF96848.1 (*V. cholerae* VpsT), AGB12693.1 (*V. parahaemolyticus* CpsQ), WP_238966351.1 (*V. alginolyticus*), WP_154184804.1 (*V. fluvialis*), WP_001189892.1 (*V. mimicus*), and WP_342651696.1 (*V. metschnikovii*).

Reference

1. Baker-Austin C, Oliver JD, Alam M, Ali A, Waldor MK, Qadri F, Martinez-Urtaza J. 2018. Vibrio spp. infections. *Nat. Rev. Dis. Primers* **4:** 8.

2. Madeira F, Pearce M, Tivey ARN, Basutkar P, Lee J, Edbali O*, et al.* 2022. Search and sequence analysis tools services from EMBL-EBI in 2022. *Nucleic Acids Res.* **50:** W276-W279.

3. Robert X, Gouet P. 2014. Deciphering key features in protein structures with the new ENDscript server. *Nucleic Acids Res.* **42:** W320-324.

4. Krasteva PV, Fong JC, Shikuma NJ, Beyhan S, Navarro MV, Yildiz FH, Sondermann H. 2010. Vibrio cholerae VpsT regulates matrix production and motility by directly sensing cyclic di-GMP. *Science* **327:** 866-868.
